# Status of potential *Pf*ATP6 molecular markers for artemisinin resistance in Suriname

**DOI:** 10.1186/1475-2875-11-322

**Published:** 2012-09-11

**Authors:** Malti R Adhin, Mergiory Labadie-Bracho, Stephen G Vreden

**Affiliations:** 1 Faculty of Medical Sciences, Department of Biochemistry, Anton de Kom Universiteit van Suriname, Kernkampweg 5, Paramaribo, Suriname; 2 “Prof. Dr. Paul C Flu” Institute for Biomedical Sciences, Kernkampweg 5, Paramaribo, Suriname; 3 Academic Hospital Paramaribo, Flu Straat, Paramaribo, Suriname

## Abstract

**Background:**

Polymorphisms within the *PfATP6* gene have been indicated as potential molecular markers for artemisinin efficacy. Since 2004, the use of artemisinin combination therapy (ACT) was introduced as first-line treatment of the uncomplicated malaria cases in Suriname. The aim of this research was to determine changes in Suriname in the status of the polymorphic markers in the *PfATP6* gene before and after the adoption of the ACT-regimen, particularly of the S769N mutation, which was reported to be associated with *in vitro* Artemether resistance in the neighboring country French Guiana.

**Methods:**

The *PfATP6* gene from *Plasmodium falciparum* parasites in Suriname was investigated in 28 samples using PCR amplification and restriction enzyme analysis, to assess and determine the prevalence of potentially interesting single nucleotide polymorphisms. The polymorphisms [L263E; A623E; S769N], which may be associated with the artemisinin resistant phenotype were characterized in parasites from three endemic regions before and after the adoption of the ACT-regimen. In addition, the status of these molecular markers was compared in paired *P. falciparum* isolates from patients with recurring malaria after controlled ACT.

**Results:**

All the investigated samples exhibit the wild-type genotype at all three positions; L263, A623, S769.

**Conclusion:**

All investigated isolates before and after the adoption of the ACT-regimen and independent of endemic region harbored the wild-type genotype for the three investigated polymorphisms. The study revealed that decreased artemisinin susceptibility could occur independent from *PfATP6* mutations, challenging the assumption that artemisinin resistance is associated with these mutations in the *PfATP6* gene.

## Background

*Artemisia annua* extracts have been used for centuries in traditional Chinese medicine, to treat febrile illnesses including malaria. Currently, artemisinin, isolated from the plant *Artemisia annua* and its synthetic derivatives are worldwide the most potent and rapidly acting anti-malarials. In 2003, the WHO recommended the use of artemisinin-based combination therapy (ACT) [1].

Parasite resistance, especially of *Plasmodium falciparum*, has been recorded to every utilized anti-malarial drug [2], which calls for a diligent monitoring of emerging artemisinin resistance in any malaria endemic area especially in light of the large parasite populations increasingly being exposed to artemisinins worldwide and the actual development of artemisinin resistance in Western Cambodia [3] and Thailand [4]. Various independent experimental approaches corroborate the emerging of artemisinin resistance: reports of cure rates below 75% after artemether-lumefantrine treatment in Cambodia [5]; the production of artemisinin-resistant strains *in vivo* in the rodent malaria model *Plasmodium yoelii *[6]; the selection of genetically stable and transmissible parasites resistant to artemisinin and artesunate in *P. chabaudi chabaudi *[7] and the observation of *in vitro* resistance to Artemether in isolates from French Guiana [8] and in travelers returning from sub-Saharan Africa [9].

These observations demonstrate that malaria parasites are not only genetically and biologically capable of sustaining stable resistance to artemisinins, but also that this resistance can be selected through drug pressure*.*

Resistance to the anti-malarial drugs pyrimethamine, sulphadoxine and chloroquine is associated with specific mutations in the *pfdhfr, pfdhps* and *pfcrt* gene, respectively. However, there is considerable controversy on the notion of mutations responsible for the emerging resistance to the class of artemisinin derived anti-malarial drugs. Various individual molecules such as haem, catalase, SERCA type enzymes have been proposed as targets for artemisinins. Several observations suggest that artemisinins inhibit the *P. falciparum* sarco–endoplasmic reticulum Ca^2+^-ATPase (SERCA), encoded by the gene denoted *PfATP6 *[10]. SERCA is a key metabolic enzyme, which reduces the cytosolic free calcium concentration.

Additional evidence that resistance to artemisinins may depend on single mutations in the SERCA-type Ca^2+^ ATPase was derived from a structure-function investigation, identifying a potential hydrophobic interaction between Leu_263_ of malarial SERCA’s with a side chain of artemisinin derivatives [11] and several studies of *in vitro* resistance to Artemether. In Senegal, one isolate was a *Pf*ATPase6 E431K/A623E double mutant [8], in sub Saharan Africa, isolates were linked to the A623E/S769N haplotype [9] and in Suriname’s neighboring country French Guiana alongside the border Maroni river, an association with the S769N substitution was revealed [8]. On the other hand, *in vivo* artemisinin resistance could not be linked to mutations in the *PfATP6* gene in clinical isolates from Cambodia [12].

The artemether-lumefantrine combination (Coartem®) was introduced in Suriname as the first-line treatment of malaria in January 2004 as part of an elaborate effort to strengthen the national malaria programme.

An active trans-border migration, especially of gold miners with high mobility and frequent use of counterfeit drugs, exists between Suriname and neighboring French Guiana, where *in vitro* resistance to Artemether was observed [8]. Although no *in vivo* resistance to artemisinin derivatives or ACT had been reported from French Guiana and artemisinin resistance was not yet clinically documented in Suriname, the circumstances called for an investigation of the potential emergence of artemisinin resistance in accordance with the WHO recommendations ‘to take urgent action to protect the efficacy of ACT’ [13].

A retrospective molecular survey was conducted to assess the status of three possible *PfATP6* molecular markers for artemisinin resistance in clinical isolates collected in the three leading endemic regions in Suriname, including the border region with French Guiana. Parasites from two different time periods in Suriname were used to gain insight in the status of the *PfATP6* gene prior to and after the introduction of ACT as first line anti-malarial drug. Furthermore, these polymorphisms of the *PfATP6* gene were analyzed in paired isolates from patients with recurring malaria after controlled ACT therapy.

## Methods

### Study site

Suriname is located along the North Coast of South America, bordering French Guiana to the east, Guyana to the west and Brazil to the south. Suriname has a population of 492.829 people (50.3% male; 49.7% female), with 49.3% living in the coastal area in and around the capital Paramaribo [14]. A savannah belt separates the narrow coastal region from the tropical rainforest of the interior, which covers 80% of the country. Malaria transmission is only observed in the interior where 9.8% of the population lives in small Maroon or Amerindian settlements.

Since the 1990s, small gold mining activities in the interior attracted about 15,000 miners mostly from Brazil and French Guiana, substantially increasing the people at risk for malaria infection.

*Anopheles darlingi* is the predominant vector for malaria transmission and *P. falciparumPlasmodium vivax* and *Plasmodium malariae* are the circulating species. In 2004, the country implemented a strengthened malaria programme with several measures, such as introduction of an ACT regimen, distribution of long-lasting impregnated nets (LLINs), use of mobile units in the mining areas and active case detection, which resulted in a spectacular decrease of 14,403 malaria cases in 2003 to 1,371 cases in 2009 [15].

### Study population

Within two independent anti-malarial drug efficacy studies, Dried Blood Spots (DBS) were collected from consenting positive patients from diverse age groups with microscopically confirmed, uncomplicated *P. falciparum* malaria. Random selections of these DBS were used for this study, 10 samples collected in 2002 and 10 samples collected in 2005. Samples were from different villages in three geographically diverse endemic regions in Suriname (*Marowijne, Boven Suriname* and *Brokopondo*).

Additionally, testing was performed on four paired filters (three from Marowijne, the border area with French Guiana) from all patients in the latter study with recurring malaria within 42 days after controlled use of ACT. The observed ACT treatment failure rate was 4.6% (n = 87) [16]. DBS from day 0 and day 42 were tested.

### DNA isolation, amplification, restriction analysis and recrudescence methods

DNA was isolated from DBS on filter paper using a modified Saponin-Chelex extraction method [17]. Primary amplification of *PfATP6* regions was followed by nested PCR with mutation specific primers to identify potential polymorphisms for both the A623E and L263E position.

For the S769N position, the flanking DNA was amplified with specifically designed primers containing an artificially introduced mismatch to generate a restriction site (*Afl*II) for the wild-type form of the two alternative alleles for position 769. Substitutions at position S769N were subsequently distinguished through enzyme digestion with *Afl*II. Primer sequences and PCR programs are described in Tables 1 and 2.

**Table 1:** Primer Sequences for PfATP6 codons 263, 623 and 769

**Genomic region**	**Primer Name**	**Primer Sequence**
**PfATP6 Codon 263**	263P1	5'-CTCCCGCTGATGCAAG-3'
	263P2	5'-CCATGAATTGGATCTG-3'
	263 N1	5'-CAGTTGACAAATATGCTG-3'
	263WT	5'-GATAATTGTTGACCAAATA-3'
	263 M	5'-GATAATTGTTGACCAAATG-3'
**PfATP6 Codon 623 and 769**	ATP1	5'-GGGTATCAACAAATTC-3'
	ATP2	5'-CTTCAAATTCTCTTCC-3'
	623WT	5'-TATACTACAGCTCAGGC-3'
	623 M	5'-TATACTACAGCTCAGGA-3'
	623 N2	5'-ACACTCATAAGTTTCC-3'
	769 N1	5'-ACTTAGCTTTGCTTATAAAAACTTAA-3'
	769 N2	5'-AATTATCCTTTTCATCATCTCC-3'

Original primer sequences from P. Cravo (personal communication).

**Table 2:** PCR programs for PfATP6 codons 263, 623 and 769

**PfATP6 Codon**	**PCR**	**Temp.**	**Time**	**N. of cycles**	**Primers**
**263**	**1**^**st **^**round PCR**	92 °C	3 min	1 cycle	263P1 and 263P2
		92 °C	30 sec	30 cycles	
		47 °C	45 sec		
		65 °C	1 min		
		68 °C	5 min	1 cycle	
		4 °C	Hold	1 cycle	
	**2**^**nd **^**round PCR**	95 °C	3 min	1 cycle	263WT and 263 N1* 263 M and 263 N1
		95 °C	45 sec	20 cycles	
		62 °C	1 min		
		72 °C	1 min		
		72 °C	10 min	1 cycle	
		4 °C	Hold	1 cycle	
**623-769**	**1**^**st **^**round PCR**	94 °C	5 min	1 cycle	ATP1 and ATP2
		94 °C	30 sec	45 cycles	
		44 °C	45 sec		
		68 °C	1 min		
		68 °C	5 min	1 cycle	
		4 °C	Hold	1 cycle	
**623**	**2**^**nd **^**round PCR**	94 °C	5 min	1 cycle	623WT and 623 N2* 623 M and 623 N2
		94 °C	30 sec	20 cycles	
		40 °C	45 sec		
		68 °C	1 min		
		70 °C	5 min	1 cycle	
		4 °C	Hold	1 cycle	
**769**	**2**^**nd **^**round PCR**	94 °C	5 min	1 cycle	769 N1 and 769 N2
		94 °C	30 sec	25 cycles	
		56 °C	45 sec		
		68 °C	1 min		
		68 °C	5 min	1 cycle	
		4 °C	Hold	1 cycle	

(*) Second round PCR for codon 263 and 623 were performed in single reactions with either the wild type (WT) or mutant (M) primer.

Recrudescence analysis and repeat number polymorphisms assessment for the four paired filters consisted of a first round PCR, followed by nested PCR for the genes GLURP, MSP-1 and MSP-2 [18,19]. Sequence polymorphisms in MSP-1 and MSP-2 were investigated through restriction analysis with *Alu*I, *Hinf*I, *Dde*I, *Scrf*I and *Rsa*I.

For all PCRs, lack of cross-contamination was monitored by the inclusion of negative control samples in each PCR-run, while the consistency of the PCR-results was confirmed through duplicate analysis of random control samples. Amplified or digested DNA products were separated through electrophoresis of polyacrylamide slab gels or horizontal agarose gels. Repeat number analysis or restriction patterns were analyzed on either high-resolution agarose or polyacrylamide slab gels in the presence of a molecular weight ladder. DNA was detected with UV-illumination after staining with ethidium bromide. Restriction efficacy in case of uncut products was monitored by the inclusion of positive control samples, which had to be completely digested, in order to convincingly ascribe “uncut” restriction patterns to the absence of the examined restriction site.

## Results

Polymorphisms in the *PfATP6* gene, encoding the SERCA-type *Plasmodium falciparum* sarco–endoplasmic reticulum Ca^2+^-ATPase, were assessed in 28 samples derived from patients with microscopically confirmed uncomplicated *P. falciparum* malaria, from three different endemic regions.

Sufficient DNA was recovered for all 28 samples and single nucleotide polymorphic assays were successfully performed for all three amino acid positions 263, 623 and 769.

The four paired samples of patients with recurring malaria after controlled treatment were all indeed recrudescent malaria isolates, as was demonstrated with repeat number and recrudescence analysis.

Position Leu_263_, was unaltered in all 28 investigated samples and none of the isolates displayed the A623E mutant genotype or the S769N mutation. It should be noted that even the recrudescent samples obtained in the border area with French Guiana still exhibited the wild-type S769 haplotype.

## Discussion

All 28 isolates exhibited an identical genotype for the three investigated positions. No changes could be detected either in samples obtained from geographical different regions or in samples collected prior to or after adopting an ACT regimen. However, all results should be regarded as indications since the sample size was small and an 18-24 months period is rather brief to exert selection pressure. The absence of the S769N mutation in clinical isolates corroborates the finding from neighboring Brazil, suggesting that the mutant S769N haplotype has probably not yet spread regionally [20].

An important finding is the observed *in vivo* decreased artemisinin susceptibility, as attested by recrudescent malaria despite monitored treatment, independent of the A623E, L263E and S769N mutations. Although the sample size was small, the results do not favor a direct association between these single nucleotide polymorphisms and resistance to artemisinin, which is supported by research on the *P. chabaudi chabaudi* ATP6 sequence revealing no nucleotide changes following selection with artesunate or artemisinin [7].

These results are also consistent with independent investigations conducted in Tanzania [21] and other African [22] and Asian [12] countries, where polymorphisms of *PfATP6* could not be linked to artemisinin resistance, but are in contrast with the findings in travelers returning from sub-Saharan Africa [9], where the A623E/S769N haplotype was linked to *in vitro* Artemether resistance. Although several samples were collected alongside the Maroni border river and even with the recurring malaria isolates originating from this area, the results from neighboring French Guiana where the S769N mutation was reported to be associated with *in vitro* Artemether resistance [8], could not be substantiated.

The finding that generation of artemisinin resistant *P. chabaudi chabaudi* clones failed in chloroquine sensitive clones and was only successful in chloroquine resistant clones [23] did not confound the results, since all investigated clones were resistant to chloroquine [Adhin, unpublished results].

In the light of accumulating evidence of artemisinin resistance slowly emerging worldwide, diligent surveillance is needed to monitor susceptibility to artemisinin derivatives in endemic areas and these results underscore the search for more appropriate molecular markers. The determination of *pfmdr1* copy number and single nucleotide polymorphisms in *pfmdr1* alongside *PfATP6* analysis might be helpful, since conflicting reports exist about the association of increased *pfmdr1* copy number and reduced susceptibility not only to Mefloquine, but also to artemisinin [9,12,24]. The use of other screening methods as monitoring parasitaemia should be considered.

## Conclusion

This report presents the first data for Suriname on single nucleotide *PfATP6* polymorphisms, potentially associated with artemisinin resistance. Isolates obtained prior to or after adopting Coartem® as first-line regimen do not exhibit any mutations at the investigated polymorphisms since all the investigated samples from Suriname harbor the wild-type genotype at all three positions; L263E, A623E, S769N. The findings in French Guiana regarding the S769N mutation could not be substantiated, despite the geographic vicinity. The occurrence of malaria relapses after controlled ACT without altering the *Pf*ATP6 genotype at the investigated positions, proved the possibility of decrease of artemisinin susceptibility independent of these mutations in the *PfATP6* gene.

## Abbreviations

ACT: Artemisinin Combination Therapy; DNA: Deoxyribonucleic Acid; DBS: Dried Blood Spots; GLURP: *P. falciparum* glutamate-rich protein (GLURP); LLIN: Long-lasting impregnated nets; MSP-1: *Plasmodium falciparum* merozoite specific protein; MSP-2: *Plasmodium falciparum* merozoite specific protein 2; PCR: Polymerase Chain Reaction; *Pf*ATP6: *Plasmodium falciparum* Ca^2+^ -ATPase protein; *PfATP6*:*Plasmodium falciparum Ca*^*2+*^*-ATPase* coding gene; *pfcrt*: *Plasmodium falciparum chloroquine resistance transporter* coding gene; *pfdhfr*:*Plasmodium falciparum dihydrofolate reductase* coding gene; *pfdhps*: *Plasmodium falciparum dihydropteroate synthase* coding gene; *pfmdr1*: *Plasmodium falciparum multidrug resistance protein 1* coding gene; *Pf*MDR1:*Plasmodium falciparum* multidrug resistance protein 1; RAVREDA: La Red Amazónica de Vigilancia de la Resistencia a los Antimaláricos (*The Amazon Network for the Surveillance of Antimalarial Drug Resistance*); SERCA: Sarco-endoplasmic reticulum Ca^2+^ - ATPase; WHO: World Health Organization.

## Competing interests

The authors declare that they have no competing interests.

## Authors’ contributions

MRA conceived of the study and participated in its design and coordination and drafted the manuscript. MLB participated in writing of the manuscript and the laboratory testing. SV participated in the design of the study and aided in the collection of the samples. All authors read and approved the final manuscript.
